# Inverse design and implementation of a wavelength demultiplexing grating coupler

**DOI:** 10.1038/srep07210

**Published:** 2014-11-27

**Authors:** Alexander Y. Piggott, Jesse Lu, Thomas M. Babinec, Konstantinos G. Lagoudakis, Jan Petykiewicz, Jelena Vučković

**Affiliations:** 1 Ginzton Laboratory, Stanford University, Stanford, CA, 94305

## Abstract

Nanophotonics has emerged as a powerful tool for manipulating light on chips. Almost all of today's devices, however, have been designed using slow and ineffective brute-force search methods, leading in many cases to limited device performance. In this article, we provide a complete demonstration of our recently proposed inverse design technique, wherein the user specifies design constraints in the form of target fields rather than a dielectric constant profile, and in particular we use this method to demonstrate a new demultiplexing grating. The novel grating, which has not been developed using conventional techniques, accepts a vertical-incident Gaussian beam from a free-space and separates O-band (1300 nm) and C-band (1550 nm) light into separate waveguides. This inverse design concept is simple and extendable to a broad class of highly compact devices including frequency filters, mode converters, and spatial mode multiplexers.

Conventional integrated photonic devices^1^ include components such as waveguide directional couplers^2^, multimode interference couplers^3^, distributed Bragg reflectors^4^, micro-ring resonators^5^, adiabatic tapers^6^ and grating couplers^7^. In all of these cases, the design space spans a relatively small (~2–5) number of parameters such as structure widths, heights and periodicity that are tuned throughout the photonic device design stage. To design a device, the photonic engineer specifies a dielectric profile, computes the electromagnetic field response using Maxwells equations, and compares the response to the device specifications. The process is then repeated, modifying the dielectric profile each iteration, until satisfactory performance is obtained. This brute force approach suffers from a long device design cycle, and does not take full advantage of the available design space of fabricable devices.

To this end, a wide variety of increasingly sophisticated approaches have been developed to search through this parameter space and optimize specific nanophotonic structures. Several methods, namely genetic algorithms^8^,^9^,^10^ and particle swarm optimization^11^, ignore the underlying physics but have achieved considerable success in fine-tuning existing structures, and designing photonic crystal devices with selectively removed holes and posts. These methods, however, are typically restricted to optimizing a relatively small number of geometric parameters, and scale poorly with additional degrees of freedom. Other methods exploit the underlying physics to quickly converge on local optima, typically by computing the local gradient of a performance metric and using steepest-descent optimization^12^. Owing to their much faster convergence, they can be used to design more complex structures with arbitrary topologies^13^,^14^,^15^,^16^,^17^,^18^,^19^. These algorithms can search through the design space of complex, aperiodic structures beyond those we can come up with based on our intuition and experience. Such devices may be able to provide novel functionality, or higher performance and smaller footprints than traditional devices, due to the greatly expanded design space.

We have recently proposed an inverse design approach for linear optical components, where the user specified input is not the basic structure of the device, but rather a set of performance metrics^19^. These include the device area, the modes of input and output waveguides to use (e.g. TE, TM, and mode order), extinction ratios (e.g. >20 dB), and insertion loss (e.g. <1 dB). More rigorously, the user only specifies the coupling efficiencies between a set of input and output modes at various frequencies. Any linear optical device can be specified in this fashion^20^, including mode converters, spatial mode multiplexers, and wavelength demultiplexers.

Two of the most important functions in integrated photonics, for both chip-to-chip and intra-chip optical interconnects, are wavelength-division multiplexing (WDM) and vertical-incidence coupling^21^. A device combining these two functions in a single, compact layout could be particularly useful for coupling between silicon photonic layers in a stacked-die microprocessor, or for coupling on/off chip using optical fibers. Although a uniform grating coupler with a tilted incident beam will act as a wavelength-demultiplexing grating coupler for two wavelength bands^22^, the symmetry imposed by a vertically incident beam implies that a uniform grating coupler cannot split wavelengths. Indeed, an efficient vertically-incident wavelength-demultiplexing grating coupler cannot be designed using current analytic methods, or by tuning a small number of parameters by hand.

Here, we provide a full demonstration of the inverse design technique's power by experimentally demonstrating a vertical-incidence, wavelength-demultiplexing grating coupler fabricated in silicon-on-insulator (SOI). The grating accepts a vertically incident beam from free space or an optical fiber, and splits O-band (~1300 nm) and C-band (~1550 nm) light into separate silicon photonic waveguides with high extinction ratios (>10 dB).

## Results

In general, we can specify the performance of a linear optical device by defining the mode conversion efficiency between a set of input modes and output modes^20^. These modes are specified by the user, and kept fixed during the optimization process. The input modes *i* = 1 … *M* are at frequencies *ω_i_*, and can be represented by equivalent current density distributions **J***_i_*. The generated electric fields **E***_i_* should satisfy Maxwell's equations in the frequency domain, 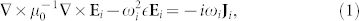
where 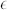 is the electric permittivity, and *μ*_0_ is the magnetic permeability of free space.

We can then specify *N_i_* output modes of interest for each input mode *i*. The output mode electric fields 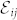 are given over output surfaces *S_ij_*, where *j* = 1 … *N_i_*. The amplitude of each output mode should be bounded between *α_ij_* and *β_ij_*, which can be expressed as 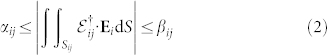
for *i* = 1 … *M* and *j* = 1 … *N_i_*. We are thus interested in finding 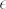 and **E***_i_* which simultaneously satisfy (1) and (2).

**Figure 1:**
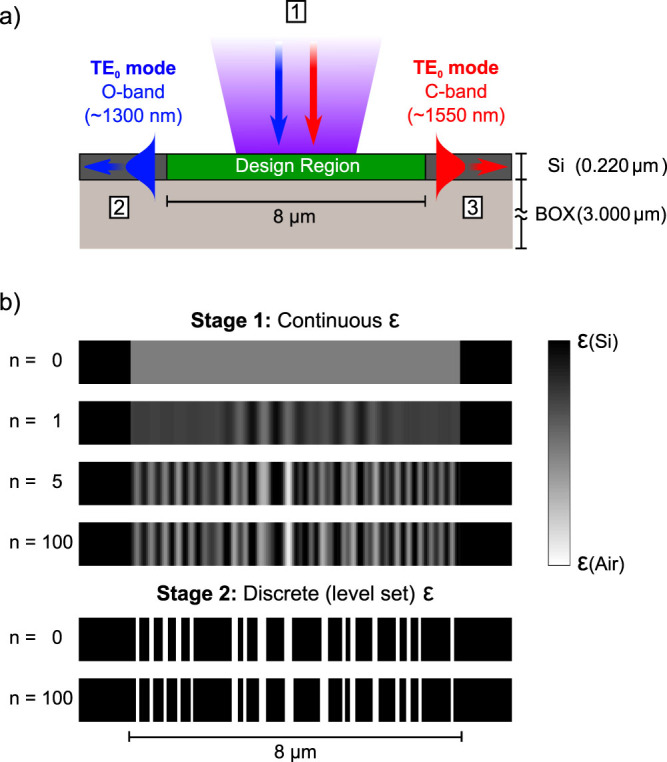
The inverse design procedure. (a) The device specifications provided to the inverse design algorithm, which consist of the input and output modes, the design region, and the surrounding structure. The device is fabricated by fully etching a 220 nm silicon layer on 3 *μ*m of buried oxide, in the pattern produced by the optimization algorithm (see figure 1b). (b) Intermediate steps in the optimization process, where *n* is the iteration number. The optimization process proceeds in two stages. In the first stage, the permittivity 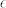 is allowed to vary continuously. In the second stage, the design is converted to a binary level-set representation and fine-tuned. To clearly illustrate the design process, the diagrams are not to scale in the vertical direction.

The WDM grating was designed by specifying the input mode to be a 4.4 *μ*m diameter vertically-incident Gaussian beam, and the output modes to be the fundamental TE mode of the output silicon slab waveguides, as shown in figure 1a. The algorithm was directed to maximize power into the left waveguide and minimize power into the right waveguide at 1300 nm, and the converse at 1550 nm.

Given the design specifications, our algorithm iteratively optimizes the structure using a close analogue of steepest-descent optimization to meet the constraints given in equation (2), as detailed in the Supplementary Information. We use finite-difference frequency domain (FDFD) simulations to calculate the local gradient during each step^23^,^24^. As illustrated in figure 1b, the design procedure consists of two stages. In the first stage, the permittivity 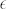 is allowed to smoothly vary within the design region. In the second stage, we convert the structure to a level-set representation^25^ and fine-tune the final structure. We did not apply any design rules such as a minimum feature size, although such constraints could be incorporated into the design process^19^. The entire inverse design process for this grating took only ~15 minutes on a single Intel Core i7 processor.

**Figure 2:**
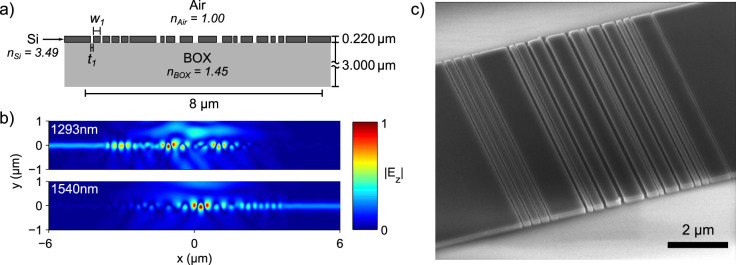
Basic structure of the wavelength demultiplexing grating. (a) The device consists of a 220 nm silicon layer with fully etched trenches on top of 3 *μ*m of buried oxide. Dimensions of the trench widths (*t_n_*) and spacings (*w_n_*), chosen by the optimization algorithm, are listed in Supplementary Table 1. (b) Frequency-domain electric field amplitudes for a 4.4 *μ*m diameter Gaussian beam incident on the center of the grating. At 1293 nm, light is only coupled into the fundamental mode of the left waveguide, whereas at 1540 nm, light is only coupled into the right waveguide. The fields plotted here were calculated using a finite-difference time-domain simulation, and post-processed using narrowband frequency filters to obtain the phasor fields. (c) Scanning electron microscopy (SEM) image of the fabricated device.

The final device was fabricated in SOI with a 220 nm thick Si device layer and a 3 *μ*m BOX (buried oxide) layer by fully etching the Si device layer, as described in figure 2a. The dimensions and locations of the grating trenches are detailed in Supplementary Table 1. The broadband performance of the device was verified by using 2D finite-difference time domain (FDTD) simulations. The phasor fields at 1293 nm and 1540 nm for a 4.4 um diameter Gaussian beam, obtained using 2D-FDTD simulations, are plotted in figure 2b. The fabricated device is presented in figure 2c.

**Figure 3:**
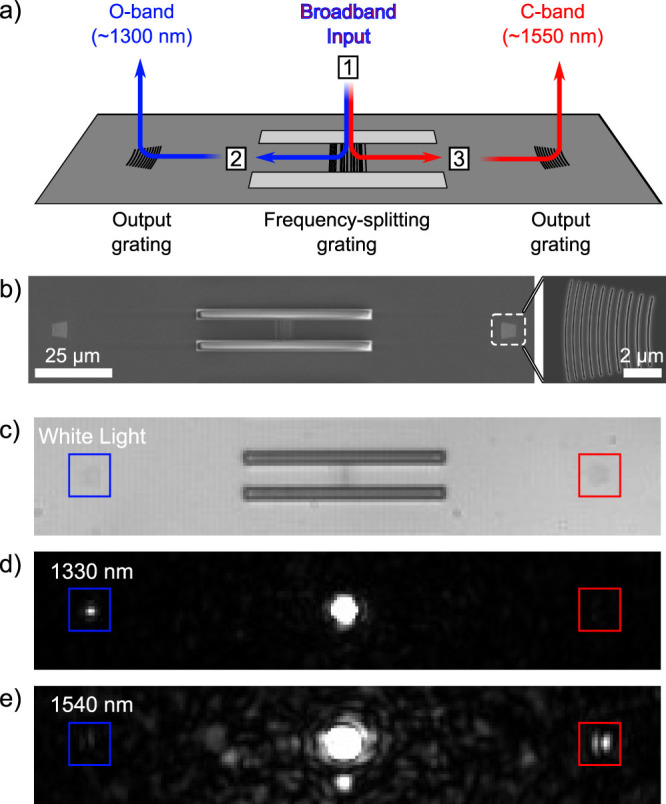
Experimental configuration used to measure the WDM grating coupler. (a) The WDM grating is excited by a free-space beam, which couples light into the silicon slab waveguide. The coupled light is then scattered upwards by the two identical output gratings, and collected via free-space optics. (b) Scanning electron microscopy (SEM) images of the fabricated structures. The wavelength-demultiplexing grating coupler is in the center of a 8 *μ*m wide, 70 *μ*m long waveguide, and the two output grating couplers are placed 50 *μ*m from the ends of the waveguide. The output grating couplers are strongly chirped to provide broadband coupling, and slightly curved to minimize back-reflections into the waveguide. (c–e) Infrared camera images of the device under (c) broad white-light illumination, and when the WDM grating is excited by a focused laser beam tuned to (d) 1320 nm and (e) 1540 nm. The locations of the two output gratings are indicated by the colored boxes. Light is only coupled to the left output grating at 1320 nm, and only the right grating at 1540 nm. Back-scatter from the rear surface of the wafer is visible near the center of both images.

The final fabricated structures also incorporate a waveguide and curved broadband output grating on either side of the wavelength-demultiplexing grating, as shown in figure 3a. Scanning electron microscopy (SEM) images of the fabricated structures are shown in figure 3b. The output gratings were strongly chirped to provide broadband performance. To minimize Fabry-Perot resonances due to back-reflections, the output gratings were slightly curved and placed far from the ends of the waveguides (inset). In future, the output waveguides could be edge-coupled to optical fibers to obtain a well-characterized out-coupling efficiency^26^.

The WDM grating was excited in the vertical direction by a focused Gaussian beam with a diameter of ~4 *μm*, and a fraction of the coupled light was out-coupled by the output gratings. To ensure a clean Gaussian input beam, the source was passed through a length of single-mode optical fiber. Spectroscopic data was measured using a broadband LED source, and narrowband images were taken using tunable lasers. The structure was both excited and measured through a single plan-apochromat microscope objective integrated into a custom confocal microscopy setup. The collected light was either directly imaged using an InGaAs near-infrared camera, or spatially filtered by a pinhole at a focal plane and analyzed using a grating spectrometer with an InGaAs detector.

Images of the device broadly illuminated with white light, and excited by a focused tunable laser are presented in figure 3c–e. At 1320 nm, light is only coupled to the left output grating, and at 1540 nm, light is only coupled to the right output grating, clearly demonstrating the basic functionality of the device.

**Figure 4:**
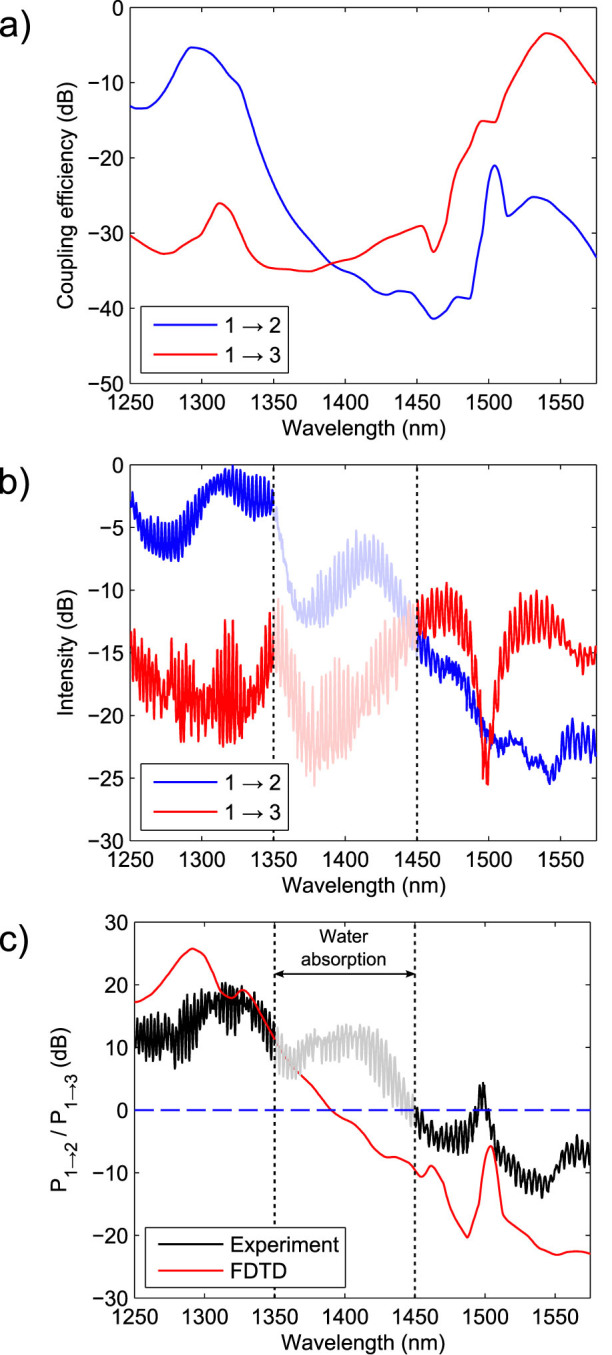
Simulated and measured coupling efficiencies of the WDM grating coupler. The device ports are labelled as in figure 3a. (a) Simulated coupling efficiency into the left and right waveguides, calculated using an finite-difference time domain (FDTD) simulation on the structure with optimized parameters. A 4.4 *μ*m diameter Gaussian beam was used as the input. (b) Experimental measured intensities from the left and right output gratings. The intensities are only normalized with respect to the source since the output grating efficiencies are not known. Around 1300 nm, light is predominantly coupled to the left grating, whereas around 1550 nm, light is predominantly coupled into the right grating. Measurements in the 1350–1450 nm band (shaded) are corrupted by water absorption lines in the atmosphere. (c) Simulated and experimentally measured splitting ratios for the coupler, defined as the the ratio of power into the left and right waveguides. We have experimentally measured splitting ratios of 17 ± 2 dB at 1310 nm and 12 ± 2 dB at 1540 nm.

In figure 4, we present both the simulated and experimentally measured coupling efficiency spectra of the WDM grating. The coupling efficiencies of the WDM grating computed using 2D FDTD are plotted in figure 4a. At 1293 nm, the simulated coupling efficiencies into the left and right waveguides are 0.2937 and 0.0008 respectively, whereas at 1540 nm, the coupling efficiencies are 0.0027 and 0.4544. The measured signal intensity, normalized to source brightness, from the two output gratings is plotted in figure 4b. The output grating coupling efficiencies were not measured, but due to the symmetry of the experimental setup, measurements of the two output ports are directly comparable. The experimental data from 1350 nm–1450 nm is shaded to due to the presence of strong atmospheric water absorption lines^27^. Fabry-perot fringing, arising from reflections between the WDM grating and output gratings, is visible in the measured spectra, with fringe spacing corresponding to the spacing between the gratings. The measured signals broadly match the simulated coupling efficiencies of the WDM grating.

The splitting ratio, defined as the ratio of power emitted from the two output ports, is plotted in figure 4c. The fabricated device has a measured splitting ratio of 17 ± 2 dB at 1310 nm and 12 ± 2 dB at 1540 nm, whereas the designed values at these wavelengths were 19.6 dB and 22.2 dB and respectively. The discrepancy between the simulated and measured splitting ratio is likely due to fabrication imperfections.

In conclusion, we have provided the first experimental demonstration of a nanophotonic device designed using our inverse design algorithm. In particular, we have implemented an efficient vertical-incidence wavelength-demultiplexing grating coupler, which cannot be designed by hand or by using parameter sweeps. By vastly opening up the parameter space for nanophotonic devices, inverse-design has broad implications for the future design of novel and compact nanophotonic components with full three-dimensional freedom.

## Methods

### Optimization algorithm and electromagnetic simulations

Our inverse design algorithm uses a close analogue of steepest-descent optimization, and incorporates the MaxwellFDFD finite-difference frequency-domain solver to calculate local gradients^23^,^24^, as detailed in the Supplementary Information.

After completing the design process, the broadband performance of the device was calculated using 2D finite-difference time-domain (FDTD) simulations.

### Fabrication

The detailed grating dimensions are listed in Supplementary Table 1. The devices were fabricated on Unibond™ SmartCut™ silicon-on-insulator (SOI) wafers obtained from SOITEC, with a nominal 220 nm device layer and 3.0 *μ*m BOX layer. A JEOL JBX-6300FS electron beam lithography system was used to pattern 330 nm of ZEP-52A electron beam resist spun on the samples. The pattern was then transferred to the Si device layer with a magnetically-enhanced reactive-ion etcher using a HBr/Cl_2_ chemistry. Finally, the mask was stripped by sonicating in Microposit remover 1165.

## Author Contributions

A.Y.P. designed and fabricated the structures, simulated their performance, and performed the experiments. J.L. developed the inverse-design algorithm used in this paper. J.V. and T.B. provided theoretical and experimental guidance, and J.V. supervised the entire project. K.G.L. and J.P. provided experimental support. All authors contributed to discussions.

## Supplementary Material

Supplementary Information Supplementary Information

## References

[b1] Sun J., Timurdogan E., Yaacobi A., Hosseini E. S. & R. Watts M. Large-scale nanophotonic phased array. Nature 493, 195–199 (2013). 23302859 10.1038/nature11727

[b2] Trinh P., Yegnanarayanan S. & Jalali B. Integrated optical directional couplers in silicon-on-insulator. Elect. Lett. 31, 2097–2098 (1995).

[b3] Soldano L. B. & Pennings E. C. M. Optical multi-mode interference devices based on self-imaging: principles and applications. J. Lightw. Technol. 13, 615–627 (1995).

[b4] Murphy T. E., Hastings J. T. & Smith H. I. Fabrication and characterization of narrow-band bragg-reflection filters in silicon-on-insulator ridge waveguides. J. Lightw. Technol. 19, 1938–1942 (2001).

[b5] Dumon P. *et al.* Low-loss SOI photonic wires and ring resonators fabricated with deep UV lithography. IEEE Photon. Technol. Lett. 16, 1328–1330 (2004).

[b6] Shani Y., Henry C. H., Kistler R. C., Kazarinov R. F. & Orlowsky K. J. Integrated optic adiabatic devices on silicon. IEEE J. Quantum Electron. 27, 556–566 (1991).

[b7] Taillaert D. *et al.* Grating couplers for coupling between optical fibers and nanophotonic waveguides. Jpn. J. Appl. Phys. 45, 6071–6077 (2006).

[b8] Gondarenko A. & Lipson M. Low modal volume dipole-like dielectric slab resonator. Opt. Express 16, 17689–17694 (2008). 18958049 10.1364/oe.16.017689

[b9] Håkansson A. & Sánchez-Dehesa J. Inverse designed photonic crystal demultiplex waveguide coupler. Opt. Express 13, 5440–5449 (2005). 19498539 10.1364/opex.13.005440

[b10] Minkov M. & Savona V. Automated optimization of photonic crystal slab cavities. Sci. Rep. 4, 5124 (2014). 24874589 10.1038/srep05124 PMC4038819

[b11] Ma Y. *et al.* Ultralow loss single layer submicron silicon waveguide crossing for SOI optical interconnect. Opt. Express 21, 29374–29382 (2013). 24514491 10.1364/OE.21.029374

[b12] Boyd S. & Vandenberghe L. Convex Optimization (Cambridge University Press, Cambridge, U.K., 2004).

[b13] Jensen J. S. & Sigmund O. Systematic design of photonic crystal structures using topology optimization: Lowloss waveguide bends. Appl. Phys. Lett. 84, 2022 (2004).

[b14] Borel P. I. *et al.* Topology optimization and fabrication of photonic crystal structures. Opt. Express 12, 1996–2001 (2004). 19475034 10.1364/opex.12.001996

[b15] Mutapcica A., Boyd S., Farjadpour A., Johnson S. G. & Avnielb Y. Robust design of slow-light tapers in periodic waveguides. Eng. Optimiz. 41, 365384 (2009).

[b16] Jensen J. S. & Sigmund O. Topology optimization for nano-photonics. Laser Photonics Rev. 5, 308–321 (2011).

[b17] Lalau-Keraly C. M., Bhargava S., Miller O. D. & Yablonovitch E. Adjoint shape optimization applied to electromagnetic design. Opt. Express 21, 21693–21701 (2013). 24104043 10.1364/OE.21.021693

[b18] Niederberger A. C. R., Fattal D. A., Gauger N. R., Fan S. & Beausoleil R. G. Sensitivity analysis and optimization of sub-wavelength optical gratings using adjoints. Opt. Express 22, 12971–12981 (2014). 24921494 10.1364/OE.22.012971

[b19] Lu J. & Vučković J. Nanophotonic computational design. Opt. Express 21, 13351–13367 (2013). 23736587 10.1364/OE.21.013351

[b20] Miller D. A. B. All linear optical devices are mode converters. Opt. Express 20, 23985–23993 (2012). 23188365 10.1364/OE.20.023985

[b21] Reed G. T. Silicon Photonics: The State of the Art (John Wiley & Sons, Chichester, West Sussex, U.K., 2008).

[b22] Roelkens G., Thourhout D. V. & Baets R. Silicon-on-insulator ultra-compact duplexer based on a diffractive grating structure. Opt. Express 15, 10091–10096 (2007). 19547358 10.1364/oe.15.010091

[b23] Shin W. & Fan S. Choice of the perfectly matched layer boundary condition for frequency-domain Maxwell's equations solvers. J. Comput. Phys. 231, 34063431 (2012).

[b24] Shin W. MaxwellFDFD webpage. (2014). URL web.stanford.edu/~wsshin/maxwellfdfd. Date of access: 2014-09-11.

[b25] Osher S. & Fedkiw R. Level Set Methods and Dynamic Implicit Surfaces (Springer, New York, U.S.A., 2003).

[b26] Kopp C. *et al.* Silicon photonic circuits: On-CMOS integration, fiber optical coupling, and packaging. IEEE J. Sel. Topics Quantum Electron. 17, 498–509 (2011).

[b27] McClatchey R. A., Fenn R. W., Selby J. E. A., Volz F. E. & Garing J. S. Optical Properties of the Atmosphere (third edition). AFCRL-72-0497 (Air Force Cambridge Research Laboratories, L. G. Hanscom Field, Bedford, Massachusetts, U.S.A., 1972).

