# Supplementary material for: Inverse design and implementation of a wavelength demultiplexing grating coupler
Source: Sci Rep. 2014 Nov 27;4:7210. doi: 10.1038/srep07210 (PMC4245525; doi:10.1038/srep07210)
Supplement: Supplementary Information [file srep07210-s1.pdf]

Supplementary Information:
Inverse design and implementation of a wavelength demultiplexing grating coupler

Alexander Y. Piggott, Jesse Lu, Thomas M. Babinec,
Konstantinos G. Lagoudakis, Jan Petykiewicz, and Jelena Vučković

1 Inverse Design Algorithm

1.1 Problem description

Maxwell's equations in the frequency domain can be written as

$$\nabla \times \mu_0^{-1} \nabla \times \mathbf{E} - \omega^2 \epsilon \mathbf{E} = -i\omega \mathbf{J} \quad (1)$$

where $\mathbf{E}$ is the electric field, $\mathbf{J}$ is the current density, ω is the frequency, ϵ is the electric permittivity, and μ_0 is the magnetic permeability of free space.

We specify the device performance by defining the mode conversion efficiency between a set of input modes and output modes. The input and output modes are specified by the user, and kept fixed during the optimization process. The input modes $i = 1 \ldots M$ are at frequencies ω_i, and can be represented by equivalent current density distributions $\mathbf{J}_i$. We can then specify N_i output modes of interest for each input mode i. The output mode electric fields $\mathcal{E}_{ij}$ are given over output surfaces S_{ij}, and the amplitude of each output mode should be bounded between α_{ij} and β_{ij}, where $j = 1 \ldots N_i$.

We are thus interested in finding ϵ and $\mathbf{E}_i$ which simultaneously satisfy

$$\nabla \times \mu_0^{-1} \nabla \times \mathbf{E}_i - \omega_i^2 \, \epsilon \, \mathbf{E}_i = -i\omega_i \mathbf{J}_i \quad (2)$$

$$\alpha_{ij} \leq \left| \iint_{S_{ij}} \mathcal{E}_{ij}^\dagger \cdot \mathbf{E}_i \mathrm{d}S \right| \leq \beta_{ij} \quad (3)$$

for $i = 1 \ldots M$ and $j = 1 \ldots N_i$. The permittivity ϵ is also subject to additional fabrication constraints.

This can be recast in the language of linear algebra by discretizing space and

making the substitutions

$$
\begin{aligned}
\mathbf{E}_i \to x_i &\in \mathcal{C}^n \\
\epsilon \to z &\in \mathcal{C}^n \\
\nabla \times \mu_0^{-1} \nabla \times \to D &\in \mathcal{C}^{n \times n} \\
-i\omega_i \mathbf{J}_i \to b_i &\in \mathcal{C}^n \\
\mathcal{E}_{ik} \to c_{ij} &\in \mathcal{C}^n.
\end{aligned} \tag{4}
$$

This leaves us with the problem

$$
D x_i - \omega_i^2 \operatorname{diag}(z) x_i - b_i = 0 \tag{5}
$$

$$
\alpha_{ij} \leq \left| c_{ij}^\dagger x_i \right| \leq \beta_{ij} \tag{6}
$$

for $i = 1 \dots M$ and $j = 1 \dots N_i$. Here, $\operatorname{diag}(v)$ refers to the diagonal matrix whose diagonal entries are given by the vector v. For convenience, we further define

$$
\begin{aligned}
A_i(z) &\triangleq D - \omega_i^2 \operatorname{diag}(z) \\
B_i(x_i) &\triangleq -\omega_i^2 \operatorname{diag}(x_i)
\end{aligned} \tag{7}
$$

which lets us rewrite equation (5) as

$$
0 = A_i(z) x_i - b_i = B_i(x_i) z + (D x_i + b_i). \tag{8}
$$

The final problem we wish to solve is then

$$
A_i(z) x_i - b_i = 0 \tag{9}
$$

$$
\alpha_{ij} \leq \left| c_{ij}^\dagger x_i \right| \leq \beta_{ij}. \tag{10}
$$

1.2 Formulating the Optimization Problem

We have previously developed two inverse design methods for designing linear optical devices: one which we call **objective-first**, and another which is an analogue of the **steepest-descent** strategy [1]. To design the WDM grating device demonstrated in this paper, we only used the steepest-descent based method, which is what we describe here.

The particular optimization problem we solve is

$$
\begin{array}{ll}
\text{minimize} & F(x_1, \dots, x_M) \\
\text{subject to} & A_i(z) x_i - b_i = 0, \quad \text{for } i = 1 \dots N \\
 & z = m(p).
\end{array} \tag{11}
$$

Here, we constrain the fields to satisfy Maxwell's equations, parameterize the permittivity z with $p \in \mathcal{R}^m$, and construct a penalty function

$$
F(x_1, \dots, x_M) = \sum_{i=1}^{M} f_i(x_i) \tag{12}
$$

for violating our field constraints from equation (10). The penalty $f_i(x_i)$ for each input mode is given by

$$f_i = \sum_{j=1}^{N_i} I_+ \left(\left| c_{ij}^\dagger x_i \right| - \alpha_{ij} \right) + I_+ \left(\beta_{ij} - \left| c_{ij}^\dagger x_i \right| \right) \tag{13}$$

where $I_+(u)$ is a relaxed indicator function [2],

$$I_+(u) = \begin{cases} 0, & u \geq 0 \\ \frac{1}{a} \left| u \right|^q, & \text{otherwise.} \end{cases} \tag{14}$$

Typically, we use $q = 2$ and $a = \max_i f_i(x_i)$.

1.3 Optimization Algorithm

We ensure that Maxwell's equations always satisfied, which implies that both the fields $x_1, \ldots, x_M$ and the field-constraint penalty function F are a function of the permittivity z. On each iteration, we locally approximate our penalty function $F(z)$ with a quadratic function

$$F(z) \approx Q(z) = \left\| Pz - q \right\|^2 \tag{15}$$

and solve the subproblem

$$\begin{array}{ll} \text{minimize} & Q(z) \\ \text{subject to} & z = m(p) \end{array} \tag{16}$$

using steepest descent optimization. The structure parameter p is sufficiently small that we can calculate the gradient $\nabla_p Q(m(p))$ using brute force. The main computational cost of the algorithm lies in computing $Q(z)$.

1.4 Choice of $Q(z)$

We choose a quadratic function $Q(z)$ of the form

$$\begin{aligned} Q(z) &= \frac{1}{2} \left\| z - z_0 \right\|^2 + \kappa \nabla_z F^\dagger(z_0) \cdot (z - z_0) \\ &= \frac{1}{2} \left\| z - (z_0 - \kappa \nabla_z F(z_0)) \right\|^2 + (\text{const.}) \end{aligned} \tag{17}$$

where z_0 is the value of z from the previous iteration in the optimization process, and $\kappa \in \mathcal{R}$. The analogy with the steepest descent strategy is clear if we consider the minimum of $Q(z)$,

$$\arg\min Q(z) = z_0 - \kappa \nabla_z F(z_0) \tag{18}$$

which is simply the steepest descent step with size κ.

We now consider how to compute the gradient $\nabla_z F$. Taking the derivative of equation (5), the discretized Maxwell's equations, with respect to z, we obtain

$$\begin{aligned} D\frac{dx_i}{dz} - \omega_i^2 \operatorname{diag}(x_i) - \omega_i^2 \operatorname{diag}(z)\frac{dx_i}{dz} &= 0 \\ \left(D - \omega_i^2 \operatorname{diag}(z)\right)\frac{dx_i}{dz} &= \omega_i^2 \operatorname{diag}(x_i) \\ A_i(z)\frac{dx_i}{dz} &= -B_i(x_i) \end{aligned} \tag{19}$$

where we have used our definitions of A_i and B_i from (7). The derivative of x_i with respect to z is then given by

$$\frac{dx_i}{dz} = -A_i^{-1}(z)B_i(x). \tag{20}$$

The gradient of the structure objective is thus

$$\nabla_z F = \sum_{i=1}^{M} \frac{d}{dz} f_i(x_i) \tag{21}$$

where

$$\frac{d}{dz} f_i(x_i) = \frac{\partial f_i}{\partial x_i}\frac{dx_i}{dz} = -\frac{\partial f_i}{\partial x_i} A_i^{-1}(z) B_i(x_i) = -\left(A_i^{-\dagger}(z)\frac{\partial f_i^\dagger}{\partial x_i}\right)^\dagger B_i(x_i). \tag{22}$$

Since A_i and B_i are large $n \times n$ matrices, we have rearranged the expression in the final step to require only a single matrix solve rather than n solves. The partial derivatives $\partial f_i / \partial x_i$ are given by

$$\frac{\partial f_i}{\partial x_i} = \sum_{j=1}^{N_i} \frac{\partial}{\partial x_i} I_+\left(\left|c_{ij}^\dagger x_i\right| - \alpha_{ij}\right) + \frac{\partial}{\partial x_i} I_+\left(\beta_{ij} - \left|c_{ij}^\dagger x_i\right|\right) \tag{23}$$

where

$$\frac{\partial}{\partial x_i} I_+\left(\left|c_{ij}^\dagger x_i\right| - \alpha_{ij}\right) = \frac{1}{2}\frac{\left(c_{ij}^\dagger x_i\right)^*}{\left|c_{ij}^\dagger x_i\right|} c_{ij}^\dagger \cdot \begin{cases} 0, & |c_{ij}^\dagger x_i| - \alpha_{ij} \geq 0 \\ \frac{q}{a}\left||c_{ij}^\dagger x_i| - \alpha_{ij}\right|^{q-1}, & \text{otherwise} \end{cases} \tag{24}$$

$$\frac{\partial}{\partial x_i} I_+\left(\beta_{ij} - \left|c_{ij}^\dagger x_i\right|\right) = \frac{1}{2}\frac{\left(c_{ij}^\dagger x_i\right)^*}{\left|c_{ij}^\dagger x_i\right|} c_{ij}^\dagger \cdot \begin{cases} 0, & \beta_{ij} - |c_{ij}^\dagger x_i| \geq 0 \\ \frac{q}{a}\left|\beta_{ij} - |c_{ij}^\dagger x_i|\right|^{q-1}, & \text{otherwise.} \end{cases} \tag{25}$$

The absolute value function $|u|$ for $u \in \mathcal{C}$ is not analytic so the complex derivative does not exist. Instead, we have used the Wirtinger derivative [3, 4] of the absolute value function in (24) - (25), which is

$$\frac{\partial}{\partial u}|u| = \frac{1}{2}\left(\frac{\partial}{\partial x} - i\frac{\partial}{\partial y}\right)|u| = \frac{u^*}{2|u|} \tag{26}$$

where we have defined $u = x + iy$ for $x, y \in \mathcal{R}$.

1.5 Parameterizing the structure

The particular structure we designed consisted of two materials in the design area, with permittivities ϵ_1 and ϵ_2. We initially used a linear parameterization of $z = m(p)$,

$$m(p) = z_{fixed} + (\epsilon_2 - \epsilon_1)\, Sp \tag{27}$$

where $S \in \mathcal{R}^{n \times m}$, $0 \leq S_{kl} \leq 1$, and $0 \leq p_k \leq 1$. The portions of the structure which were kept fixed during the optimization process were described by $z_{fixed} \in \mathcal{C}$.

In the second step of the optimization, we converted to a level set representation of the structure using thresholding. When constructing z, we took care to apply anti-aliasing to the borders of the structure.

1.6 Solving Maxwell's equations using FDFD

We must efficiently solve Maxwell's equations in the frequency domain at the following points in our algorithm:

1. Evaluating the fields x_i at the beginning of each iteration.
2. Solving the adjoint problem in equation (22) to compute the gradient $\nabla_z F$ of the field-constraint penalty.

This was done by using the MaxwellFDFD package for MATLAB [5, 6].

2 WDM grating dimensions

n	t_n (nm)	w_n (nm)
**1**	81.5	235.4
**2**	83.4	235.5
**3**	73.5	243.5
**4**	81.9	218.8
**5**	61.1	928.3
**6**	147.1	85.2
**7**	78.7	331.8
**8**	164.4	450.8
**9**	204.6	603.2
**10**	192.6	257.0
**11**	68.2	139.0
**12**	130.6	399.5
**13**	139.1	362.8
**14**	98.5	156.8
**15**	105.2	163.0
**16**	75.0	665.7
**17**	64.4	—

Supplementary Table 1: List of parameters for the grating coupler. The trench widths (t_n) and spacings (w_n) are indicated in figure **2a** of the main manuscript.

References

[1] Lu, J. & Vučković, J. Nanophotonic computational design. *Opt. Express* **21**, 13351 – 13367 (2013).

[2] Boyd, S. & Vandenberghe, L. *Convex Optimization* (Cambridge University Press, Cambridge, U.K., 2004).

[3] Gunning, R. C. & Rossi, H. *Analytic functions of several complex variables*. Prentice-Hall series in modern analysis (Prentice-Halls, Englewood Cliffs, New Jersey, U.S.A., 1965).

[4] Petersen, K. B. & Pedersen, M. S. *The Matrix Cookbook*. Technical University of Denmark (2012).

[5] Shin, W. & Fan, S. Choice of the perfectly matched layer boundary condition for frequency-domain Maxwell's equations solvers. *J. Comput. Phys.* **231**, 34063431 (2012).

[6] Shin, W. MaxwellFDFD webpage (2014). URL `web.stanford.edu/~wsshin/maxwellfdfd`. Date of access: 2014-09-11.
